# South African pre-hospital emergency care personnel’s lived experiences of managing paediatric emergencies: A qualitative research design utilising one-on-one interviews

**DOI:** 10.4102/hsag.v26i0.1558

**Published:** 2021-07-22

**Authors:** Colin G. Mosca, Christopher Stein, Heather Lawrence

**Affiliations:** 1 Department of Emergency Medical Care, Faculty of Health Science, University of Johannesburg, Johannesburg, South Africa; 2 Department of Allied Health Professions, Faculty of Health and Applied Sciences, University of the West of England, Bristol, United Kingdom

**Keywords:** paediatrics, emergencies, South Africa, pre-hospital emergency care, emergency care personnel, perceptions, lived experiences

## Abstract

**Background:**

The phenomenon of managing paediatric emergencies in the pre-hospital environment within the South African setting is poorly understood with specific regard to what emergency care personnel are experiencing when managing paediatric emergencies.

**Aim:**

The aim of this study was to explore and describe the lived experiences of emergency care personnel in managing paediatric patients in the pre-hospital environment and to understand the meaning and the significance of these experiences.

**Setting:**

All participants were purposively sampled from emergency medical services agencies operating within the Johannesburg metropolitan city area.

**Methods:**

This study followed a qualitative, exploratory, descriptive, phenomenological design, whereby participants purposively sampled within the Johannesburg metropolitan city voluntarily consented to one-on-one interviews (*n* = 10).

**Results:**

Three main themes, with 11 contributing categories, were identified and contextualised with available literature. Emerging from the main themes was an overall sense that managing paediatric emergencies is a negative experience, coloured with feelings of inadequacy, stress, anxiety and even fear.

**Conclusions:**

The findings of this study provided new insights into what South African EMS are experiencing when managing paediatric emergencies, which enables future research efforts to identify research and practice gaps that are relevant to paediatric pre-hospital emergency care, and that are specific to the South African environment.

**Contribution:**

This research provides preliminary insight into the lived experiences of prehospital personnel managing paediatric emergencies as well as emerging recommendations for the improvement of the prehospital care of paediatric patients.

## Introduction

The emergency medical services (EMS) play an important role in patient care, which must include the provision of good-quality care and robust patient safety mechanisms. These are ever-evolving qualities that need to be actively implemented and maintained as the EMS system evolves (Hoyle et al. 2012; Meckler, Leonard & Hoyle 2014). Numerous studies have found that paediatric patients are generally not provided with the same level of care as adult patients within emergency care settings when presenting with similar injury profiles requiring similar clinical management strategies (Ardolino et al. 2012; Breon et al. 2011; Browne et al. 2016a; Houston & Pearson 2010; Murphy et al. 2014; Wyen et al. 2010).

One of the largely unexplored aspects of paediatric emergency care, within the South African EMS setting, is the human component. Understanding what EMS personnel are experiencing when managing paediatric emergencies will provide direction for the purpose of future research initiatives into this aspect of South African EMS function.

In terms of the functions of EMS, there are a plethora of unique challenges that inhibit and undermine the actualisation of improving paediatric pre-hospital care in the EMS environment. These include a lack of standardisation of protocols, lack of pre-hospital patient safety systems, highly volatile and stressful work environment and an overall lack of system support (Breon et al. 2011; Guise et al. 2017; Meckler et al. 2014; Schmidt et al. 2016).

Africa has been identified as the ‘least healthiest’ place to live (Ademuyiwa et al. 2012). What this means is that Africa shoulders the highest burden of mortality as a result of trauma, communicable diseases and non-communicable diseases, whilst conversely being the most under-resourced continent (specifically in terms of healthcare provision) on the planet (Ballot et al. 2016; Kuzma et al. 2015; Reynolds et al. 2017). The sub-Saharan African region hosts the highest burden of paediatric trauma and injury emergencies with 95% of the global burden occurring in this region and 50% of children aged 0–6 years dying within the pre-hospital environment (Purcell et al. 2017).

South Africa has a trauma death rate of six times the global rate, with road traffic accidents being the leading cause of death in the age group of 5–14 years (Saggie 2013). It has been observed that the paediatric population is disproportionately affected in terms of morbidity and mortality compared to global trends, with an injury rate of 3.89/100 000 to 9.33/100 000, thus presenting itself as a major public health concern within South Africa (Manchev et al. 2015; Skinner et al. 2015). Furthermore, various South African studies have identified that current pre-hospital and emergency care for the paediatric patient in the South African context is sub-optimal, with a vast deficit in available, locally generated research (Ballot et al. 2016; Manchev et al. 2015; Purcell et al. 2017; Skinner et al. 2015).

The illustrated disparity in the appropriateness of pre-hospital emergency care provided to children promotes the idea that research into the management of paediatric emergencies would play an integral role in providing some answers to addressing this disparity in care. Although research in the area of paediatric pre-hospital emergency care has been conducted for a number of years, this research has several limitations in its South African application because of the fact that the majority of this research has been conducted within high-income settings. Research appears to be focused on topics reflecting researchers’ preferences resulting in a significant disparity between what is researched versus what should be researched (Browne et al. 2016a).

Consequently, research of an exploratory and inductive nature that is focused on the experiences of emergency care personnel who manage paediatric emergencies in the pre-hospital environment was required to understand the important, contextual problems that EMS face. There are a number of professional pre-conceptions surrounding the emergency care of paediatric patients, and the experiences therein, but there has been no formal enquiry into what the range and shared meaning of these experiences are, and what the potential consequences of these experiences might be on patient care. The aim of this study was to explore and describe the lived experiences of emergency care personnel in managing paediatric patients in the pre-hospital environment and to understand the meaning and the significance of these experiences.

## Research methods and design

### Study design

This study followed a qualitative, exploratory, descriptive, phenomenological design. This approach was used to explore, describe and understand the meaning of common themes identified for the phenomenon that was studied. Given that the study was borne out of the realisation that there was very little relevant data available about the process of managing paediatric emergencies in the pre-hospital environment within the South African setting, it became self-evident that the use of a phenomenological design was the most appropriate research design for this study (Poggenpoel, Myburgh & Linde 2006). It has been established that effective quantitative research efforts rely heavily on successful qualitative enquiry, and research efforts dealing with people-centred problems. An initial approach based on a qualitative methodology allows for a more natural, truthful discovery and identification of the research problem, which then provides the platform for focused, relevant and useful further quantitative enquiry (Poggenpoel et al. 2006).

### Setting

The setting of the Johannesburg metropolitan city was chosen as the area is diverse. It consists of urban, peri-urban and rural communities, as well as a vast number of displaced communities in the form of various informal settlements. As a result, emergency care personnel working in this area are exposed to a diverse range of paediatric emergencies, specifically with regard to epidemiology.

### Study population and sampling strategy

This study was conducted in Johannesburg, South Africa, in the metropolitan city area with purposive sampling of operational emergency care personnel (Table 1) who were invited to participate through the researcher’s professional network and contacts, as well as through participant referral post-interview processes. Participants were interviewed *when they were* off duty, at a time and venue most convenient to them - which consisted of residences and secluded places chosen by the participants. In terms of the purposive selection of the participants, the inclusion criteria were applied in conjunction with the researcher’s intent to obtain a heterogeneous sample (Table 1) with regard to sampling over a large geographical, ethnic and gender base, to promote the trustworthiness of the data (Creswell 2007; Noy 2008; Shenton 2004). The inclusion criteria were emergency care personnel with a minimum of 1 year’s clinical experience, with a reported exposure to paediatric calls and who were willing to describe their experiences for research purposes. There is a wide range of pre-hospital qualifications and scopes of practice; however, a brief overview, relevant to this study, of this may provide better clarity within an international context, which is provided in Table 2 (Sobuwa & Christopher 2019).

**TABLE 1:** Demographical characteristics of participants.

Participant	Gender	Race	Qualification	Years qualified	Sector	Johannesburg Area
1	Male	White	ECP	2	Private	West and North
2	Female	White	ECP	2	Private	Central and North
3	Female	Black	CCA	1 1/5	Public	South and Central
4	Female	Black	CCA	1 1/5	Public	South and West
5	Male	White	AEA	10	Private	North and West
6	Female	Black	AEA	10	Public	South and West
7	Female	White	AEA	15	Private	Central and South and West
8	Male	White	AEA	3	Public	North and West & South
9	Male	White	AEA	15	Public	North and South & West
10	Female	Black	BAA	4	Public	North and Central

ECP, emergency care practitioner; CCA, critical care assistant; AEA, ambulance emergency assistant; BAA, basic ambulance assistant.

These are the registration categories for emergency care personnel held with the Health Professions Council of South Africa, with the accompanying accepted abbreviations.

**TABLE 2:** Overview of emergency medical services qualifications pertaining to this study.

Qualification	General scope of practice	Autonomy in paediatric care	Paediatric-specific scope
Emergency care practitioner (ECP)	Intensive and critical care and advanced life support	Independent	Intensive care and advanced life support
Critical care assistant (CCA)	Advanced life support	Independent, with support	Advanced life support
Ambulance emergency assistant (AEA)	Intermediate life support	Supervised	Basic life support under 8 years or limited intermediate life support above 8 years
Basic ambulance assistant (BAA)	Basic life support	Supervised	Basic life support

### Data collection

The researchers explored the phenomenon through one-on-one, in-person interviews that were voice-recorded. The interviews were conducted by the first author, who prepared with reviewing acceptable interview practices, and underwent evaluation post the first interview by a seasoned interviewer. The interviews were unstructured; only one formatted question was posed so that participants were able to freely lead the process of sharing their own experiences, thus allowing for unbridled exploration into the phenomenon. Given that participants were recruited through the researcher’s professional network, good rapport was enjoyed during the interview processes. The question that was posed to the participants was ‘How is it for you to manage paediatric emergencies in the pre-hospital environment?’. The interview technique and method adopted by the researcher were specifically employed to facilitate deep and wide explorations into the experiences shared by the participants, with regular probing questions and statement reflections to encourage comfortable dialogue and participant openness throughout the interview (Creswell 2007; Qu & Dumay 2011; Shenton 2004). The researcher observed data saturation as the point where additional data sets no longer offered new insights or perspectives along the line of enquiry of the experience of managing paediatric emergencies in the prehospital environment, and this began occurring between the fifth, sixth and seventh interview processes (Anney 2014; Guest, Bunce & Johnson 2006; Celliers 2009; Charmaz 2006; Fusch & Ness 2015; Shenton 2004). Whilst collecting data, the researcher reflected on each interview by writing field notes and started the analysis process by confirming the content with each participant (Celliers 2009; Shenton 2004). The field notes consisted of the researcher’s observations of participants during the interview process-capturing non-verbal cues, emotional expressions and physical actions or reactions of participants during the interview process (Celliers 2009; Creswell 2007; Shenton 2004).

### Data analysis

The ‘Data Analysis Spiral’ approach described by Creswell was how the researcher engaged with and structured the data analysis phase of the research project (Creswell 2014). This process that was followed to analyse the interview data collected for this study included (1) transcribing interviews and sorting field notes, (2) organising, ordering and sorting the data and (3) repeatedly listening to and reading the collected material (Halloway & Wheeler 2013). The interviews were transcribed by the researcher, and this was performed immediately post-completion of each interview process and prior to the initiation of the next interview process. Furthermore, data analysis was performed manually by the researcher throughout the analysis process.

### Measures of trustworthiness

The precepts of credibility, transferability, dependability and confirmability were applied to ensure the trustworthiness of this research. The manner in which this was practically performed is detailed in Table 3 (Creswell 2007, 2014; Shenton 2004).

**TABLE 3:** Trustworthiness measures.

Strategy	Criteria
Credibility	The researchers ensured triangulation of data using in-depth, individual interviews and field notes written during data collection, and the researcher’s positioning prior to data collection. Member checking, independent re-coding and ongoing reflection were conducted. An audit trail was composed by the researcher, consisting of the researcher’s journaling, field notes and transcriptions
Transferability	The background information and contextual elements relevant to this study were explored and described in relation to emergency care personnel. The applicable settings were provided and described in detail to promote an assessment of transferability of findings. The method of sampling, data collection and analysis has been described in detail to promote reader evaluation for transferability of the research.
Dependability	The researchers recorded a detailed description of data gathering, analysis and interpretation referenced against accepted practices, which included data triangulation, independent re-coding and purposive sampling. These activities also generated an audit trail available for verification.
Confirmability	The researchers ensured triangulation of data (in-depth interviews, field notes and researcher positioning), while also ensuring member checking and ongoing reflection throughout data gathering and analysis. These activities also generated an audit trail available for verification

The setting of the study promotes the trustworthiness because of the diversity within the Johannesburg metropolitan area and the impact this has on the variety of experiences that EMS personnel have and can subsequently share in the interview process. This allowed for a greater transferability of the results to other urban low- to middle-income countries settings (Anney 2014; Cooper & Endacott 2007; Shenton 2004). Table 3 elaborates on the steps taken to ensure trustworthiness in the form of credibility, transferability, dependability and confirmability.

### Ethical considerations

Ethical approval for this research was obtained from the Faculty of Health Sciences Research Ethics Committee of the University of Johannesburg with clearance number REC-01-23-2017. Only once ethical clearance was granted did the study commence with fieldwork and data collection. All purposively sampled emergency care personnel were required to provide written informed consent before participating in this research. During the initial explanation, the researcher emphasised that participants could withdraw consent at any time during the research without any consequences to them and were free to disengage from the interview at any time without consequence. Participants were required to provide written informed consent before participating in this research. No explicit risks or benefits were identified. During data collection, confidentiality was ensured by conducting interviews in a neutral and private location, as selected by the participants. The transcriptions for each participant were annotated with pseudonyms to ensure participant anonymity and confidentiality (Creswell 2007).

### Results

A total of 10 participants were interviewed, with the use of purposive sampling in reference to the selection criteria as well as in order to obtain a heterogeneous sample. Table 1 describes the characteristics of the sample.

The data yielded three main themes that were composed of a total of 11 categories, as demonstrated in Table 4. The themes are presented below through the voices of the participants and contextualised with available relevant literature. Through reviewing local and international literature, it would appear that the phenomenon of managing paediatric emergencies produces similar experiences in EMS personnel from various systems around the world. This is significant as it indicates that the experiences shared by the participants are both authentic and relatable.

**TABLE 4:** Description of themes and associated categories.

Theme	Category
Paediatric calls make you feel helpless	Paediatric calls are different There is a lack of system support Managing parental pain is difficult Paediatric calls are more demanding
Are we doing enough?	Shortage of paediatric-specific equipment Lack of paediatric-specific facilities Need for paediatric-specific training
Paediatric calls stay with you	Avoidable injuries or deaths cause angry feelings Paediatric cases are emotionally stressful Going the extra mile for paediatrics Heightened emotional responses towards paediatrics

#### Theme 1: Paediatric calls make you feel helpless

The experiences shared by the participants evoked a strong sense of disempowerment and overwhelming circumstances that ultimately fostered an environment fraught with seemingly insurmountable challenges. The shared experience emerging within the group of participants was that the combined effect of external factors that impact the evolution and the experience of a paediatric call made them feel helpless, and in some instance even hopeless. A participant explained:

‘Ja (Yes) … sometimes … but ah … I think … ja (yes) powerless, yes … but ah, for a moment … but there’s always that little thing that actually knocks you back on track and says hey … get yourself together and you need to do this for this … for the sake of this child … you really need to get your act together …’ (Participant 6, female, public sector)

**Category 1: Paediatric calls are different:** The participants spoke about how the limited exposure to paediatric calls and the subsequent infrequent practice of skills and competencies associated with treating paediatrics contributed to a sense of overarching unfamiliarity and a feeling of perceived inadequacy in terms of clinical performance. Participants’ said:

‘I haven’t practiced some of the skills that I was equipped with.’ (Participant 4, female, public sector)

‘… kids (Children) I haven’t done too many of them …’ (Participant 8, male, public sector)

‘… managing, managing it’s … how can I explain it to you … cause, at the moment … we don’t get a lot of pead (Paediatric), pead (Paediatric) patients … ’ (Participant 9, male, public sector)

**Category 2: There is a lack of system support:** The participants described how they feel unsupported in managing paediatric calls, as they experienced that there was a lack of or no system support that they could rely on when managing a paediatric emergency. Participants relayed:

‘Um, but then; one by one, the systems that are supposed to support you, don’t allow for support in the environment …’ (Participant 1, male, private sector)

‘There’s no um … in term … the system doesn’t have contingencies, that’s what … that’s how I’ll put it … the system doesn’t have contingencies, that’s the problem …’ (Participant 2, female, private sector)

The lack of protocols also featured in the participants’ descriptions around the lack of support in managing paediatric emergencies, as shown in the following participant statement:

‘no protocol … no nothing what so ever …’ (Participant 3, female, public sector)

**Category 3: Managing parental pain is difficult:** A common experience shared by the participants was having to deal with parents and families during a paediatric emergency, and the difficulties associated with this encounter. During the participants sharing, there appeared to be a relationship between experiencing the emotional load of the incident and clinically performing, as demonstrated in the following quotations:

‘… its mom, dad, both sets of grandparents, cousins, aunts, uncles and everyone trying to get involved and make a decision and … um … just get in the way.’ (Participant 5, male, private sector)

‘she was crying unbelievably and you know, it’s difficult when you now have to try now treat the patient, console the mother.’ (Participant 2, female, private sector)

‘then you don’t want to do something and then the mom questions you a million times, and asks you what are doing this, and why are doing this, and what is this for … or what is that for and then …’ (Participant 8, male, public sector)

**Category 4: Paediatric calls are more demanding:** The participants indicated how the burden of care is not always equally shared and that this impacted personnel comfort levels in terms of getting involved. This process of carrying the bulk of the clinical load may promote the belief that paediatric calls are more demanding for high-acuity emergency care personnel (top tier paramedics), whilst low-acuity emergency care personnel (middle and bottom tier paramedics) experience pressure related to being incapable of responding to the needs of the situation:

‘so there’s all this decision making that has to take place, in like a millisecond … which is again … you know … a lot on a person’s plate …’ (Participant 2, female, private sector)

‘when it’s kids (Children) and it’s serious I’m just a bystander.’ (Participant 8, male, public sector)

‘you know, you get a call, you go, you assess the child … put them in the ambulance, there’s not much you can do … take them to hospital … there’s nothing we can give.’ (Participant 7, female, public sector)

Participants mentioned feeling that paediatric patients require greater clinical acumen, as compared with adult patients, and that this would impact how they manage paediatric emergencies. This feeling of inadequacy alone creates a basis for participants to feel that paediatric calls are more demanding. Participants reflected:

‘… makes you feel worse because you already felt you didn’t manage the patient well, you were way over your head …’ (Participant 1, male, private sector)

‘… environment for the child, I know it’s not the best that I could’ve given, but this is what I had. You know and ja (Yes) …’ (Participant 4, female, public sector)

#### Theme 2: Are we doing enough?

Participants reflected on various dynamics associated with rendering care in a functional manner. A sentiment started to emerge that participants felt that paediatrics were neglected. By observation, it became very apparent that emergency care personnel are acutely aware of persisting shortfalls in their capacity to render emergency care to paediatric patients. When sharing their experience, there appeared to be evolving criticisms and acknowledgements that demonstrated a body of professionals who appear to be lodged between practicality and ideology in terms of what they actually do, and what they know they should do. This was specifically captured by one participant as shown in the quotation below:

‘Um … I don’t want to expose the, our department but in terms of a paediatrics we don’t have much … um … but ah … I so wish that we, we could have quite a lot of ah … knowledge, as well as equipment regarding the, ah … the, paediatrics … it’s like they are neglected … and ah …’ (Participant 6, female, public sector)

**Category 1: Shortage of paediatric-specific equipment:** Participants consistently described a shortage of paediatric-specific equipment in their working environments. They also shared how this impacted the way they were able to manage paediatric emergencies, as described below:

‘… aah … so do you do do you have the correct equipment so for instance aah, so for instance um, I know, I know they say aah no you can use, you can use a feeding tube to aah, to cannulate the, to cannulate the … the umbilicus. I’m gonna say its bull (Untrue); it’s a it is incredibly difficult to do it because the diameter is too large so … of the feeding tube.’ (Participant 1, male, private sector)

‘you know and getting to hospital you get nailed (Get into Trouble) for not having an incubator, eh … why are you using bottles (Hot water bottles), you have to actually explain all the process that I am pre-hospital and pre-hospitably I sometimes just have to … um … come up with plans to make it a suitable …’ (Participant 4, female, public sector)

‘… sometimes it’s as bad as even having a BVM (Bag Valve Mask), an adult, using an adult BVM (Bag Valve Mask) … ah, we don’t have ah pead’s (Paediatric) BVM (Bag Valve Mask) … um and we are forced to work in those conditions …’ (Participant 6, female, public sector)

**Category 2: Lack of paediatric-specific facilities:** The participants explained how non-specialist facilities were often reluctant to accept paediatric emergencies, and when they did it appeared as if they were incapable of ensuring and maintaining an adequate continuum of care. Participants felt frustrated and incapacitated in terms of getting the paediatric patient to the right paediatric capable or dedicated facility:

‘Like if you do a trauma patient, a trauma child facilities’ is a nightmare. Because the area that we are in … X does not cope with trauma very well, and then X doesn’t take kids (Children), X doesn’t have any trauma facility and then it’s a nightmare cause you’re now going out of area to try fight with X to accept and the distance … distance with a child is far.’ (Participant 7, female, private sector)

‘… and another thing, with hospitals again … it’s a, it’s a challenge … going to a hospital, I like okay fine … because we got demarcations … so I’ve got a paediatric, where do I go … you go to X, they chase you away.’ (Participant 3, female, public sector)

**Category 3: Need for paediatric-specific training:** The overwhelming message expressed by all the participants was that emergency care personnel want to receive more training on paediatric emergency care, as they believe that it will improve their experience when managing paediatric emergencies. Furthermore, the participants felt that additional training might reduce the amount of stress and anxiety experienced on paediatric calls, given the perception that emergency care personnel feel out of practice as described below:

‘… blatantly I feel we don’t have enough, um … training or ja (Yes) … knowledge, so it very very limited, from what I recall on AEA (Ambulance Qualification), it’s a very small section.’ (Participant 5, male, private sector)

‘I don’t know how other people feel … but when we did our training … we never spent any time in the neonatal … clinic … or … treating any patients, small babies or infants …’ (Participant 9, male, public sector)

‘… they don’t know what to do … got a paediatric … they don’t know, so, it’s, it’s one of those things … so if training was actually emphasised … they lack in many things.’ (Participant 3, female, public sector)

#### Theme 3: Paediatric calls stay with you

A common experience shared by all the participants was one of emotional pain and, to varying degrees, psychological trauma rooted in the emotional experiences surrounding paediatric calls. Apparently, there is a significant emotional investment in paediatric calls that seems to have far-reaching effects. Some participants said that they still experience nightmares or emotional responses linked to emotionally traumatising paediatric calls. A participant explained:

‘just tell you when you do kids … kids (Children) stay with you … You get moss (you know) that calls, that calls that will always stay with you forever … kids (Children) stay with you … especially they are … it’s like I said, if it’s a result of an adult … mistake, then that kid (Child) will stay with you, especially if the adult was not harmed … so if it’s an MVA (Motor Vehicle Collision) and the other driver was intoxicated and he’s walking around on scene shouting and screaming in the meantime there’s a family dead the kids (Children) are dead … that stays with you.’ (Participant 8, male, public sector)

**Category 1: Avoidable injuries or deaths cause angry feelings:** The participants expressed feelings of anger and frustration as a result of attending paediatric calls where the injury or death of a child was the result of parental or caregiver negligence. This is emphasised in the following excerpts:

‘… you are mad at the people, or at the parents… because … they, they … should have realised, not to leave a baby alone for that long and then … why not make sure that the babies safe, or keep an eye on her … Ah … I don’t know if maybe it’s a cultural thing … maybe we are overprotective as parents … cause, I was, I was like that … my son was born I was every 5 minutes by the cot hey.’ (Participant 9, male, public sector)

‘… sorry to use the word, but it, it, it actually pissed me off … when a, um … a, a peads (Paediatric) walked in with burns, cause I failed to understand how, how did it happen … and in most cases, when you interview the parent or the person who brought the child you realise that it’s negligence from the, the parents …’ (Participant 6, female, public sector)

**Category 2: Paediatric cases are emotionally s tressful:** The sentiment that paediatric calls are emotional and stressful was a common experience shared by the participants. Strong feelings of fear, anxiety, guilt and regret were fielded during the participants’ accounts of their experiences. As the participants described their experiences, there was a vivid undertone that emergency care personnel have a far greater fear of failure on paediatric calls versus adult calls. An example of this fear is evident in the following responses:

‘I think it was difficult because you tend to … stay in the background, not want to get involved … because I think it’s of a kiddy (Child), and, and child … ah … and you don’t want to have that … bond … I don’t think you want to have … if something happens, it’s my fault … then you want to blame yourself then …’ (Participant 9, male, public sector)

‘Well, the first, like, key word is stressful. Incredibly stressful … And also it depends on the age of the child as well … between toddler and sort of the … towards the end of the toddler phase; there I’ve had a couple of emergencies. And I find that those are the most stressful.’ (Participant 2, female, private sector)

‘… and from there, ah, I think you kind of carry that with you. Um, so the second patient I had, there was a lot more anxiety and stress related because of the first experience.’ (Participant 1, male, private sector)

**Category 3: Going the extra mile for paediatrics:** The participants shared various experiences that supported the underlying reality that emergency care personnel’s work ethic on paediatric calls is emotionally driven. In the descriptions given, it would appear that there is a general greater emotional commitment to paediatric patients that translates into an increased sense of responsibility to achieve a positive outcome in managing paediatric calls. Participants relayed:

‘… with me I will go an extra mile, I will fight tooth and nail for paediatrics …’ (Participant 6, female, public sector)

‘… kids, I’ll always give them a fighting chance … regardless of what the back story is, when it happened, how it happened … I’ll give them a chance … and um you know, it’s the least you can do … especially for, for children …’ (Participant 2, female, private sector)

‘I, I think that you going out your way, to do more for them. In a sense that you will rather help them trying to do extra, go the extra mile for you, trying to save them … um … make sure that he’s safe and get him to hospital …’ (Participant 9, male, public sector)

**Category 4: Heightened emotional responses t owards paediatrics:** Participants experienced paediatric emergencies as seemingly singularly rooted in the fact that the patients were children. This was strongly demonstrated in terms of managing a paediatric death versus an adult death, regardless of whether or not any actual patient management was required. The following participant responses illustrate this connection:

‘And I think it’s just, that it’s a young life … it’s a young life, it had … it still had lots to do …’ (Participant 7, female, private sector)

‘… it’s one of those, it’s not like an adult, I would say, ag it’s an adult is outlived … it’s fine (laughs) … it’s fine, it was bound to happen … at least he’s had plus, minus years … With a kid (Child), it’s, its different …’ (Participant 3, female, public sector)

‘… I suppose not for us all … kids (Children)is … why’s it so bad to go declare (Confirm Death) a kid (Child) and you are like ja (Yes) … it’s just another p4 (Deceased patient) when you go to declare an adult …’ (Participant 8, male, public sector)

## Discussion

### Theme 1: Paediatric calls make you feel helpless

#### Category 1: Paediatric calls are different

Managing paediatric patients is a unique experience that is associated with having its own challenges and quirks. In fact, some researchers have gone so far as to say that ‘children are not small adults’ (Ademuyiwa et al. 2012; Alisic et al. 2017; O’Leary et al. 2014). The significance of this relates to the views expressed by participants who give the impression that managing paediatric patients is an unfamiliar experience that is different from regular experiences in the work environment (managing adult patients).

Globally, the norm is that paediatric calls are seen infrequently, resulting in a low exposure rate (Ardolino et al. 2012; Brown et al. 2016b; O’Leary et al. 2014; Schmidt et al. 2016; Wyen et al. 2010). Essentially, EMS personnel are not comfortable with paediatric patients because they do not manage them often (Alisic et al. 2017; Guise et al. 2017; O’Leary et al. 2014). Rooted in the feeling of unfamiliarity is the personnel’s own self-awareness of the low exposure rate; simply put, they feel out of practice (Alisic et al. 2017; Guise et al. 2017; Haghparast-Bidgoli et al. 2010; Hoyle et al. 2016; Lammers, Byrwa & Fales 2012; O’Leary et al. 2014).

#### Category 2: There is a lack of system support

The negative effects of a poor or non-existent referral network have been demonstrated on a global front, always ending with the same conclusion; increased mortality rates (Ademuyiwa et al. 2012; Ballot et al. 2016; Calvello et al. 2013; Kuzma et al. 2015). This is no different in South Africa, where there is a lack of referral systems and paediatric-specific facilities and networks available for the EMS to approach when dealing with paediatric emergencies (Hanewinckel et al. 2010; Skinner et al. 2015).

Internationally, it is accepted that there is a lack of standardisation in paediatric pre-hospital care, and that greater attention needs to be paid to the development of guidelines (protocols) aimed at improving practice (Chafe, Harnum & Porter 2016; Ebben et al. 2013; Gausche-Hill et al. 2014; Hoyle et al. 2012; Martin-Gill et al. 2016; Meckler et al. 2014; Tiyyagura et al. 2017). South Africa deals with a similar situation, with there being a lack of paediatric-specific guidelines to guide EMS personnel.

#### Category 3: Managing parental pain is difficult

It has been demonstrated that paediatric calls are emotionally charged with higher levels of anxiety and stress (Alisic et al. 2017; Falgiani, Kennedy & Jahnke 2014; Guise et al. 2017). When the emergency care personnel are confronted with the emotional experience of the injured child’s parents and family, it causes additional pressure and stress. Personnel can be affected by the emotionality of the experience, to the point where it negatively affects patient safety (Guise et al. 2017).

#### Category 4: Paediatric calls are more demanding

Various studies indicated how the common practice in EMS staffing models results in one provider playing multiple clinical care roles simultaneously, which has been seen to ultimately increase error rates (Rappaport et al. 2016; Schmidt et al. 2016).

Various studies have indicated that the younger the child, the higher the error rate by EMS personnel, and that practice errors in children tend to have a worse effect on outcome compared to adults (Guise et al. 2017; Jain & Guglani 2016; Lerner et al. 2017). This feeling of inadequacy alone creates a basis for participants to feel that paediatric calls are more demanding. This experience is also seen in EMS personnel across the globe (Alisic et al. 2017; Browne et al. 2016b).

### Theme 2: Are we doing enough?

#### Category 1: Shortage of paediatric-specific equipment

Globally, the availability and proper use of paediatric-specific equipment has been identified as insufficient to render care; multiple studies have concluded that this persisting reality is proving to be a barrier to good-quality care (Butler & Adefuye 2019; Gausche-Hill et al. 2014; Hoyle et al. 2016; Meckler et al. 2014; Nair et al. 2014). In fact, a number of studies have identified that a lack of paediatric-specific equipment available to EMS personnel is a patient safety issue. It creates increased error rates on the part of EMS personnel in rendering care in a paediatric emergency (Hagiwara et al. 2016; Lammers et al. 2012; Ramachandra et al. 2017).

The situation in South Africa is no different, given the experiences shared by the participants and the findings of various local studies. These indicated the lack of availability of appropriate resources and equipment to deliver general healthcare for simple healthcare needs, let alone care for a ‘minority demographic’ (Ballot et al. 2016; Benatar 2013; Butler & Adefuye 2019; Lala et al. 2014; Skinner et al. 2015).

#### Category 2: Lack of paediatric-specific facilities

In various countries, the lack of paediatric-specific facilities is so severe, it has been regarded as a barrier to good-quality care and a contributor to increased morbidity and mortality rates (Falgiani et al. 2014; Hanewinckel et al. 2010; Lala et al. 2014). A particular South African study looked at an area with a population of 3 million people and found that of the 23 healthcare facilities, none were dedicated to paediatric care, and only two had dedicated paediatric units (Manchev et al. 2015). A number of South African studies concluded that increased availability of facilities and networks dedicated to paediatrics within South Africa would significantly reduce mortality rates (Ballot et al. 2016; Lala et al. 2014; Manchev et al. 2015).

#### Category 3: Need for paediatric-specific training

On a global scale, it is widely believed that the ‘range and depth’ of paediatric-focused training and education provided for EMS personnel is insufficient to support the complex functions required for these healthcare professionals (Guise et al. 2017; Hansen et al. 2016; Lim et al. 2013; Kaufmann et al. 2018; Reynolds et al. 2017). The local condition is similar with various local studies identifying the significant knowledge and competency gaps present in EMS personnel; specifically pertaining to paediatric emergency care, leading to recommendations of improved training and education in this domain (Calvello et al. 2013; Hanewinckel et al. 2010; Saggie 2013; Manchev et al. 2015; Ballot et al. 2016; Butler & Adefuye 2019).

The majority of EMS personnel would like to receive additional, focused paediatric training, believing that it will improve their competency and overall performance (Alisic et al. 2017; Butler & Adefuye 2019; Chafe et al. 2016; Falgiani et al. 2014; Hoyle et al. 2016).

### Theme 3: Paediatric calls stay with you

#### Category 1: Avoidable injuries or deaths cause angry feelings

Limited research has been conducted regarding the impact that witnessing child abuse has on first responders and emergency care personnel. The few studies that have been conducted found that emergency care personnel witnessing child abuse or negligence have the potential to be emotionally and psychologically effected in a catastrophic way with various consequences (Fallat 2014; Guise et al. 2017; Ranse & Arbon 2008). These studies cite issues like burnout and disruptions in clinical performance as being the most common effects; however, a number of other consequences have also been suggested. Further research into this phenomenon may prove useful in promoting and supporting mental health and wellness in emergency care personnel (Fallat 2014; Guise et al. 2017; Ranse & Arbon 2008).

#### Category 2: Paediatric cases are emotionally stressful

Working with paediatric patients is accepted as being emotionally stressful, producing heightened levels of anxiety, and in some cases fear (Alisic et al. 2017; Hansen et al. 2016; Murphy et al. 2014; Skinner et al. 2015). Some studies have even established a link between high levels of stress and anxiety and disrupted performance in terms of rational thinking and clinical discrimination (Fallat 2014).

The effects are well documented and have been described as impairing memory recall, reducing EMS personnel’s situational awareness, producing tunnel vision and impairing decision-making abilities (Falgiani et al. 2014; Guise et al. 2017; Hansen et al. 2016; O’Leary et al. 2014). Patient safety initiatives focused on pre-hospital paediatric care have gone so far as to indicate that the fear-producing emotional process is a cause for error and a contributing barrier to good-quality paediatric care (Guise et al. 2017; Hansen et al. 2016).

#### Category 3: Going the extra mile for paediatrics

Various studies, focusing on paediatric cardiac arrest, identified how the increased psychological and cognitive load associated with these cases diminishes the capacity of healthcare workers to objectively and rationally terminate resuscitation efforts at appropriate times, leading to the situation of ‘fruitless’ and unwarranted extraordinary efforts (Fallat 2014; Guise et al. 2017; Ranse & Arbon 2008). This arises from an emotional connection; participants revealed that it is harder to accept that a child has died, and this frequently leads to feelings of guilt and failure.

#### Category 4: Heightened emotional responses towards paediatrics

The significance is that regardless of the many variables that interplay in any emergency situation, and despite the circumstances of the EMS personnel, there will always be an innate human response to the suffering or death of a child to varying degrees. While there is no research that these authors could find that directly investigates this phenomenon, it does appear to feature as an undertone in many of the studies cited and, of course, in the results of this research. Further research aimed at understanding this phenomenon may provide insights that could prove useful in better preparing emergency care personnel for the phenomenon of managing paediatric emergencies.

## Recommendations

Stemming from the experiences shared by the participants, four recommendations were formulated with the intention of highlighting key focus areas for future research initiatives. The recommendations largely echo the concerns shared by the participants in the form of suggested research activities that would begin the process of addressing their concerns.

### Training needs of pre-hospital staff

The first recommendation is to investigate and identify the current training needs of pre-hospital staff in the management of paediatric emergencies. Additional training initiatives aimed at standardising the approach to managing paediatric emergencies that are applicable across all levels of qualifications would significantly improve the standard of pre-hospital paediatric care. It would be invaluable to identify what areas of paediatric emergency care pre-hospital staff feel inadequately trained to manage, as well as management areas that appear to be consistently sub-optimally performed. The usefulness of this kind of understanding would play a vital role in directing educational initiatives aimed at addressing identified persisting shortfalls in pre-hospital personnel’s education and practice.

### The status of the local emergency medical services system

The second recommendation is to define and describe the actual status of the South African EMS system with regard to the specific management of paediatric emergencies. Identifying the actual status of the EMS system would enable the development of a national standard that would help to ensure standardisation with regard to equipment, training and overall resource capacitation, perhaps contained within the ambits of an acute referral system. The role of effective acute care referral systems tailored to the specific needs of the relevant patient demographics has been shown to reduce mortality and morbidity. There is no apparent standard that persists in the pre-hospital environment in the management of paediatric emergencies within the South African setting, and addressing this shortcoming would improve paediatric pre-hospital emergency care.

### Psychological and emotional impact of attending paediatric emergencies

The third recommendation is to investigate and define the acute and long-term psychological and emotional impact of attending paediatric emergencies, with the specific purpose of developing sustainable coping mechanisms for pre-hospital personnel within the South African setting. Fundamentally, it would appear that pre-hospital staff are not currently equipped to respond to the needs of this component of a paediatric emergency. This area of pre-hospital care would benefit from upskilling and personnel capacitation through structured debriefing interventions and support networks to help emergency care personnel deal with the emotional and psychological effects associated with managing paediatric emergencies. This activity needs to be prioritised as it would ultimately improve the quality of care provided to the patient. The overall cognitive burden exerted by the emotionality and stress of a paediatric emergency would be modulated as staff would be able to better handle the emotional and psychological impact.

### Local incidence of paediatric emergencies

The fourth recommendation is to investigate and identify the actual South African incidence of paediatric emergencies within the EMS system, and understand the demographics and associated variations in the rate of incidence. Understanding these demographics allows for the inception and development of a set of locally relevant and applicable solutions that facilitate a targeted response to a specifically defined problem – paediatric emergencies. The formulation of workable and relevant policies and strategies relies on a ‘good fit’ with the problems they are intended to resolve. With policymaking, applicable initiatives can be designed in responding to problems that have been specifically identified and scoped. This benefit further extends to the provision of required educational initiatives aimed at addressing the probable shortfalls that are likely to manifest as a result of this kind of research.

## Limitations

The sample size reflects the point of reaching data saturation. It remains unclear how scalable these results are in terms of wide spread transferability. Perhaps, with multicentre or multiple site studies, a greater degree of generalisability and transferability may be achieved; however, it may not yield any difference in the point of data saturation demonstrated in this study.

## Conclusion

The findings of the study were comparable to similar studies that have been conducted in various parts of the world, with some contextual differences related to the specific circumstances of the South African setting. The most significant contribution of this study is based in the fact that it is the first study of its kind to be conducted within the South African setting.

Previous understandings about what pre-hospital personnel were experiencing when managing paediatric emergencies was based on the undocumented sharing of anecdotal experiences leading to widely accepted and untested assumptions within the prehospital domain. The value of this research is rooted in the fact that the results are a documented through a rigorous research process, meaning that they are an authentic, real-world representation of what is being experienced by emergency care personnel within the South African setting.

Whilst globally the advancement of paediatric pre-hospital emergency care and research has been identified, there is scant research that can position the South African environment in this global agenda. The findings of this study enable future research efforts to identify actual existing research and practice gaps relevant to the South African setting, meaning that efforts aimed at improving paediatric pre-hospital emergency care can be tailored to meet the needs specific to the South African environment.

## References

[CIT0001] Ademuyiwa, A.O., Usang, U.E., Oluwadiya, K.S., Ogunlana, D.I., Glover-Addy, H., Bode, C.O. et al., 2012, ‘Pediatric trauma in sub-Saharan Africa: Challenges in overcoming the scourge’, *Journal of Emergencies, Trauma, and Shock* 5(1), 55–61. 10.4103/0974-2700.93114 PMC3299155 22416156

[CIT0002] Alisic, E., Tyler, M.P., Giummarra, M.J., Kassam-Adams, R., Gouweloos, J., Landolt, M.A. et al., 2017, ‘Trauma-informed care for children in the ambulance: International survey among pre-hospital providers’, *European Journal of Psychotraumatology* 8(1). 10.1080/20008198.2016.1273587 PMC5328382 28326162

[CIT0003] Anney, V.N., 2014, ‘Ensuring the quality of the findings of qualitative research: Looking at trustworthiness criteria’, *Journal of Emerging Trends in Educational Research and Policy Studies* 5(2), 272–281.

[CIT0004] Ardolino, A., Cheung, C.R., Sleat, G.K.J. & Willet, K.M., 2012, ‘Regional networks for children suffering major trauma’, *Emergency Medicine Journal* 29(5), 349–352. 10.1136/emermed-2011-200915 22389348

[CIT0005] Ballot, D.E., Davies, V.A., Cooper, P.A., Chirwa, T., Argent, A. & Mer, M., 2016, ‘Retrospective cross-sectional review of survival rates in critically ill children admitted to a combined paediatric/neonatal intensive care unit in Johannesburg, South Africa, 2013–2015’, *BMJ Open* 6(6), e010850. 10.1136/bmjopen-2015-010850 PMC4893876 27259525

[CIT0006] Benatar, S.R., 2013, ‘The challenges of health disparities in South Africa’, *South African Medical Journal* 103(3), 154–155. 10.7196/SAMJ.6622 23472690

[CIT0007] Breon, A. , Yarris, L., Law, J. & Meckler, G., 2011, ‘Determining the paediatric educational needs of prehospital providers: part 1’, *Journal of Paramedic Practice* 3(8), 510–514. 10.12968/jpar.2011.3.8.450

[CIT0008] Browne, L.R., Shah, M.I., Studnek, J.R., Farrell, B.M., Mattrisch, L.M., Reynolds, S. et al., 2016a, ‘2015 pediatric research priorities in prehospital care’, *Prehospital Emergency Care* 3127(August), 1–6. 10.3109/10903127.2015.1102997 26808233

[CIT0009] Browne, L.R., Shah, M.I., Studnek, J.R., Ostermayer, D.G., Reynolds, S., Guse, C.E. et al., 2016b, ‘Multicenter evaluation of prehospital opioid pain management in injured children’, *Prehospital Emergency Care* 20(6), 759–767. 10.1080/10903127.2016.1194931 27411064

[CIT0010] Butler, M.W. & Adefuye, A.O., 2019, ‘Assessing the knowledge of emergency medical care personnel in the Free State, South Africa, on aspects of paediatric pre-hospital emergency care’, *Pan African Medical Journal* 32, 1–13. http://www.panafrican-med-journal.com/content/article/32/98/full/10.11604/pamj.2019.32.98.17718 PMC6560997 31223388

[CIT0011] Calvello, E., Reynolds, T., Hirshon, J.M., Buckle, C., Moresky, R., O’Neill, J. et al., 2013, ‘Emergency care in sub-Saharan Africa: Results of a consensus conference’, *African Journal of Emergency Medicine* 3(1), 42–48. 10.1016/j.afjem.2013.01.001

[CIT0012] Celliers, B., 2009, ‘Book review: Introducing qualitative research in psychology’, *Evaluation Journal of Australasia* 9(2), 54–55. 10.1177/1035719x0900900210

[CIT0013] Chafe, R., Harnum, D. & Porter, R., 2016, ‘Improving the treatment and assessment of moderate and severe pain in a pediatric emergency department’, *Pain Research and Management* 2016, Art. ID 4250109. 10.1155/2016/4250109 PMC5031872 27672348

[CIT0014] Charmaz, K., 2006, *Constructing grounded theory: A practical guide through qualitative analysis*, in D. Silverman (ed.), Goldsmiths College, Sage. SAGE Publications Inc., Thousand Oaks, California

[CIT0015] Cooper, S. & Endacott, R., 2007, ‘Generic qualitative research: A design for qualitative research in emergency care?’, *Emergency Medicine Journal* 24(12), 816–819. 10.1136/emj.2007.050641 18029510 PMC2658349

[CIT0016] Creswell, J.W., 2007, *Qualitative inquiry & research design*, 3rd edn., Sage, Thousand Oaks, California

[CIT0017] Creswell, J.W., 2014, *Research design*, 4th edn., Sage, Thousand Oaks, California

[CIT0018] Ebben, R.H.A., Vloet, L.C.M., Verhofstad, M.H.J., Meijer, S., Mintjes-de Groot, J.A.J. & Van Achterberg, T., 2013, ‘Adherence to guidelines and protocols in the prehospital and emergency care setting: A systematic review’, *Scandinavian Journal of Trauma, Resuscitation and Emergency Medicine* 21(1), 1–16. 10.1186/1757-7241-21-9 PMC3599067 23422062

[CIT0019] Falgiani, T., Kennedy, C. & Jahnke, S., 2014, ‘Exploration of the barriers and education needs of non-pediatric hospital emergency department providers in pediatric trauma care’, *International Journal of Clinical Medicine* 05(02), 56–62. 10.4236/ijcm.2014.52011

[CIT0020] Fallat, M.E., 2014, ‘Withholding or termination of resuscitation in pediatric out-of-hospital traumatic cardiopulmonary arrest’, *Annals of Emergency Medicine* 63(4), 504–515. 10.1016/j.annemergmed.2014.01.013 24655460

[CIT0021] Fusch, P.I. & Ness, L.R., 2015, ‘Are we there yet? Data saturation in qualitative research’, *The Qualitative Report* 20(9), 1408–1416.

[CIT0022] Gausche-Hill, M., Brown, K.M., Oliver, Z.J., Sasson, C., Dayan, P.S., Eschmann, N.M. et al., 2014, ‘An evidence-based guideline for prehospital analgesia in trauma’, *Prehospital Emergency Care* 18(Suppl. 1), 25–34. 10.3109/10903127.2013.844873 24279813

[CIT0023] Guest, G., Bunce, A., Johnson, L., 2006, ‘How Many Interviews Are Enough?: An Experiment with Data Saturation and Variability’, *Field Methods* 18, 59–82. 10.1177/1525822X05279903

[CIT0024] Guise, J., Hansen, M., O’Brien, K., Dickinson, C., Meckler, G., Engle, P. et al., 2017, ‘Emergency medical services responders’ perceptions of the effect of stress and anxiety on patient safety in the out-of-hospital emergency care of children: A qualitative study’, *BMJ Open* 7(2), 1–6. 10.1136/bmjopen-2016-014057 PMC5337745 28246139

[CIT0025] Haghparast-Bidgoli, H., Hasselberg, M., Khankeh, H., Khorasani-Zavareh, D. & Johansson, E., 2010, ‘Barriers and facilitators to provide effective pre-hospital trauma care for road traffic injury victims in Iran: A grounded theory approach’, *BMC Emergency Medicine* 10(1), 20. 10.1186/1471-227X-10-20 21059243 PMC2992044

[CIT0026] Hagiwara, M.A., Nilsson, L., Strömsöe, A., Axelsson, C., Kängström, A. & Herlitz, J., 2016, ‘Patient safety and patient assessment in pre-hospital care: A study protocol’, *Scandinavian Journal of Trauma, Resuscitation and Emergency Medicine* 24(1), 1–7. 10.1186/s13049-016-0206-7 PMC4751749 26868416

[CIT0027] Halloway, I. & Wheeler, S., 2013, *Qualitative research in nursing and healthcare*, 3rd edn., John Wiley & Sons. West Sussex, United Kingdom

[CIT0028] Hanewinckel, R., Jongman, H.P., Wallis, L.A. & Mulligan, T.M., 2010, ‘Emergency medicine in Paarl, South Africa: A cross-sectional descriptive study’, *International Journal of Emergency Medicine* 3(3), 143–150. 10.1007/s12245-010-0185-9 21031037 PMC2926869

[CIT0029] Hansen, M., Meckler, G., Lambert, W., Dickinson, C., Dickinson, K., Van Otterloo, J. 2016, ‘Patient safety events in out-of-hospital paediatric airway management: A medical record review by the CSI-EMS’, *BMJ Open* 6(11), e012259. 10.1136/bmjopen-2016-012259 PMC5128842 27836871

[CIT0030] Houston, R. & Pearson, G.A., 2010, ‘Ambulance provision for children: A UK national survey’, *Emergency Medicine Journal* 27(8), 631–636. 10.1136/emj.2009.088880 20515914

[CIT0031] Hoyle, J.D., Davis, A.T., Putman, K.K., Trytko, J.A., & Fales, W.D., 2012, ‘Medication dosing errors in pediatric patients treated by emergency medical services’, *Prehospital Emergency Care* 16(1), 59–66. 10.3109/10903127.2011.614043 21999707

[CIT0032] Hoyle, J.D., Sleight, D., Henry, R., Chassee, T., Fales, B. & Mavis, B., 2016, ‘Pediatric prehospital medication dosing errors: A mixed-methods study’, *Prehospital Emergency Care* 20(1), 117–124. 10.3109/10903127.2015.1061625 26400075

[CIT0033] Jain, S. & Guglani, V., 2016, ‘Patient safety in paediatrics and neonatal medication’, *Jmr* 2(1), 16–19.

[CIT0034] Kaufmann, J., Roth, B., Engelhardt, T., Lechleuthner, A., Laschat, M., Hadamitzky, C. et al., 2018, ‘Development and prospective federal state-wide evaluation of a device for height-based dose recommendations in prehospital pediatric emergencies: A simple tool to prevent most severe drug errors’, *Prehospital Emergency Care* 22(2), 252–259. 10.1080/10903127.2016.1248257 27925849

[CIT0035] Kuzma, K., Lim, A.G., Kepha, B., Nalitolela, N.E. & Reynolds, T.A. 2015, ‘The Tanzanian trauma patients’ prehospital experience: A qualitative interview-based study’, *BMJ Open* 5(4), e006921–e006921. 10.1136/bmjopen-2014-006921 PMC4420946 25916487

[CIT0036] Lala, S.G., Britz, R., Botha, J. & Loveland, J., 2014, ‘Paediatric liver transplantation for children treated at public health facilities in South Africa: Time for change’, *South African Medical Journal* 104(11), 829–832. 10.7196/SAMJ.8624 26038799

[CIT0037] Lammers, R., Byrwa, M. & Fales, W., 2012, ‘Root causes of errors in a simulated prehospital pediatric emergency’, *Academic Emergency Medicine* 19(1), 37–47. 10.1111/j.1553-2712.2011.01252.x 22251191

[CIT0038] Lerner, E.B., Drendel, A.L., Cushman, J.T., Badawy, M., Shah, M.N., Guse, C.E. et al., 2017, ‘Ability of the physiologic criteria of the field triage guidelines to identify children who need the resources of a trauma center’, *Prehospital Emergency Care* 21(2), 180–184. 10.1080/10903127.2016.1233311 27710155

[CIT0039] Lim, C.A.E., Kaufman, B.J., O’Connor, J. & Cunningham, S.J., 2013, ‘Accuracy of weight estimates in pediatric patients by prehospital emergency medical services personnel’, *American Journal of Emergency Medicine* 31(7), 1108–1112. 10.1016/j.ajem.2013.04.018 23706580

[CIT0040] Manchev, V., Bruce, J.L., Oosthuizen, G.V., Laing, G.L., & Clarke, D.L., 2015, ‘The incidence, spectrum and outcome of paediatric trauma managed by the Pietermaritzburg Metropolitan Trauma Service’, *Annals of the Royal College of Surgeons of England* 97(4), 274–278. 10.1308/003588414X14055925061595 26263934 PMC4473864

[CIT0041] Martin-Gill, C., Gaither, J.B., Bigham, B.L., Myers, J.B., Kupas, D.F. & Spaite, D.W., 2016, ‘National prehospital evidence-based guidelines strategy: A summary for EMS stakeholders’, *Prehospital Emergency Care* 20(2), 175–183. 10.3109/10903127.2015.1102995 26808116

[CIT0042] Meckler, G., Leonard, J. & Hoyle, J., 2014, ‘Pediatric patient safety in emergency medical services’, *Clinical Pediatric Emergency Medicine* 15(1), 18–27. 10.1016/j.cpem.2014.01.003

[CIT0043] Murphy, A., Barrett, M., Cronin, J., McCoy, S., Larkin, P., Brenner, M. et al., 2014, ‘A qualitative study of the barriers to prehospital management of acute pain in children’, *Emergency Medicine Journal* 31(6), 493–498. 10.1136/emermed-2012-202166 23520269

[CIT0044] Nair, M., Yoshida, S., Lambrechts, T., Boschi-Pinto, C., Bose, K., Mason, E.M. et al., 2014, ‘Facilitators and barriers to quality of care in maternal, newborn and child health: A global situational analysis through metareview’, *BMJ Open* 4(5) 1–19. 10.1136/bmjopen-2013-004749 PMC4039842 24852300

[CIT0045] Noy, C., 2008, ‘Sampling knowledge: The hermeneutics of snowball sampling in qualitative research’, *International Journal of Social Research Methodology* 11(4), 327–344. 10.1080/13645570701401305

[CIT0046] O’Leary, F., McGarvey, K., Christoff, A., Major, J., Lockie, F., Chayen, G. et al., 2014, ‘Identifying incidents of suboptimal care during paediatric emergencies-an observational study utilising in situ and simulation centre scenarios’, *Resuscitation* 85(3), 431–436. 10.1016/j.resuscitation.2013.12.001 24321323

[CIT0047] Poggenpoel, M., Myburgh, C.P. & Van der Linde, C., 2006, ‘Qualitatve research strategies as prerequisite for quantitative strategies.pdf’, *Education* 122(2), 408–412.

[CIT0048] Purcell, L., Mabedi, C. E., Gallaher, J., Mjuweni, S., McLean, S., Cairns, B. et al., 2017, ‘Variations in injury characteristics among paediatric patients following trauma: A retrospective descriptive analysis comparing pre-hospital and in-hospital deaths at Kamuzu Central Hospital, Lilongwe, Malawi’, *Malawi Medical Journal* 29(2), 146–150. 10.4314/mmj.v29i33 28955423 PMC5610286

[CIT0049] Qu, S.Q. & Dumay, J., 2011, ‘The qualitative research interview’, *Qualitative Research in Accounting & Management* 8(3), 238–264. 10.1108/11766091111162070

[CIT0050] Ramachandra, G., Shepherd, M., Shepherd, C.M. & Child, S., 2017, *Symposium Article Year : 2017 | Volume : 4 | Issue : 1 | Page : 24-29 Golden Hour Management of Pediatric Polytrauma in India- Emergency Department Resuscitation Correspondence* Address. Intensive Care Chapter of India Academy of Pedaitrics, New Delhi.

[CIT0051] Ranse, J. & Arbon, P., 2008, ‘Graduate nurses’ lived experience of in-hospital resuscitation: A hermeneutic phenomenological approach’, *Australian Critical Care* 21(1), 38–47. 10.1016/j.aucc.2007.12.001 18206382

[CIT0052] Rappaport, L.D., Brou, L., Givens, T., Mandt, M., Balakas, A., Roswell, K. et al., 2016, ‘Comparison of errors using two length-based tape systems for prehospital care in children’, *Prehospital Emergency Care* 20(4), 508–517. 10.3109/10903127.2015.1128027 26836351 PMC6292711

[CIT0053] Reynolds, T.A., Stewart, B., Drewett, I., Salerno, S., Sawe, H.R., Toroyan, T. et al., 2017, ‘The impact of trauma care systems in low- and middle-income countries’, *Annual Review of Public Health* 38(1), 507–532. 10.1146/annurev-publhealth-032315-021412 28125389

[CIT0054] Saggie, J., 2013, ‘Trauma: South Africa’s other epidemic’, *South African Medical Journal* 103(9), 589–590. 10.7196/SAMJ.7387

[CIT0055] Schmidt, A.R., Ulrich, L., Seifert, B., Albrecht, R., Spahn, D.R. & Stein, P., 2016, ‘Ease and difficulty of pre-hospital airway management in 425 paediatric patients treated by a helicopter emergency medical service: A retrospective analysis’, *Scandinavian Journal of Trauma, Resuscitation and Emergency Medicine* 24(1), 1–8. 10.1186/s13049-016-0212-9 PMC4779199 26944389

[CIT0056] Shenton, A.K., 2004, ‘Strategies for ensuring trustworthiness in qualitative research projects’, *Education for Information* 22(2), 63–75. 10.3233/EFI-2004-22201

[CIT0057] Skinner, D.L., Den Hollander, D., Laing, G.L., Rodseth, R.N. & Muckart, D.J., 2015, ‘Severe blunt thoracic trauma: Differences between adults and children in a level I trauma centre’, *South African Medical Journal* 105(1), 47–51. 10.7196/SAMJ.8499 26046163

[CIT0058] Sobuwa, S. & Christopher, L.D., 2019, ‘Emergency care education in south africa: Past, present and future’, *Australasian Journal of Paramedicine* 16, 1–5. 10.33151/ajp.16.647

[CIT0059] Tiyyagura, G.K., Gawel, M., Alphonso, A., Koziel, J., Bilodeau, K. & Bechtel, K., 2017, ‘Barriers and facilitators to recognition and reporting of child abuse by prehospital providers’, *Prehospital Emergency Care* 21(1), 46–53. 10.1080/10903127.2016.1204038 27436455

[CIT0060] Wyen, H., Jakob, H., Wutzler, S., Lefering, R., Laurer, H.L., Marzi, I. et al., 2010, ‘Prehospital and early clinical care of infants, children, and teenagers compared to an adult cohort: Analysis of 2,961 children in comparison to 21,435 adult patients from the trauma registry of DGU in a 15-year period’, *European Journal of Trauma and Emergency Surgery* 36(4), 300–307. 10.1007/s00068-010-1124-4 26816034

